# Molecular Phylogeny of OVOL Genes Illustrates a Conserved C2H2 Zinc Finger Domain Coupled by Hypervariable Unstructured Regions

**DOI:** 10.1371/journal.pone.0039399

**Published:** 2012-06-21

**Authors:** Abhishek Kumar, Anita Bhandari, Rahul Sinha, Puspendu Sardar, Miss. Sushma, Pankaj Goyal, Chandan Goswami, Alessandro Grapputo

**Affiliations:** 1 Department of Biology, University of Padua, Padova, Italy; 2 Abteilung für Botanische Genetik und Molekularbiologie, Botanisches Institut und Botanischer Garten, Christian-Albrechts-Universität zu Kiel, Kiel, Germany; 3 Lehrstuhl für Molekulare Phytopathologie, Christian-Albrechts-Universität zu Kiel, Kiel, Germany; 4 Cold Spring Harbor Laboratory, Cold Spring Harbor, New York, United States of America; 5 National Institute of Science Education and Research, Institute of Physics Campus, Sachivalaya Marg, Bhubaneswar, Orissa, India; 6 Indian Institute of Toxicology Research, Lucknow, Uttar Pradesh, India; 7 Klinik und Poliklinik für Frauenheilkunde und Geburtshilfe, Universitätsklinikum, Der Ernst-Moritz-Arndt-Universität Greifswald, Greifswald, Germany; University of Wyoming, United States of America

## Abstract

OVO-like proteins (OVOL) are members of the zinc finger protein family and serve as transcription factors to regulate gene expression in various differentiation processes. Recent studies have shown that OVOL genes are involved in epithelial development and differentiation in a wide variety of organisms; yet there is a lack of comprehensive studies that describe OVOL proteins from an evolutionary perspective. Using comparative genomic analysis, we traced three different OVOL genes (OVOL1-3) in vertebrates. One gene, OVOL3, was duplicated during a whole-genome-duplication event in fish, but only the copy (OVOL3b) was retained. From early-branching metazoa to humans, we found that a core domain, comprising a tetrad of C2H2 zinc fingers, is conserved. By domain comparison of the OVOL proteins, we found that they evolved in different metazoan lineages by attaching intrinsically-disordered (ID) segments of N/C-terminal extensions of 100 to 1000 amino acids to this conserved core. These ID regions originated independently across different animal lineages giving rise to different types of OVOL genes over the course of metazoan evolution. We illustrated the molecular evolution of metazoan OVOL genes over a period of 700 million years (MY). This study both extends our current understanding of the structure/function relationship of metazoan OVOL genes, and assembles a good platform for further characterization of OVOL genes from diverged organisms.

## Introduction

Hierarchical and extremely diverse sets of transcriptional regulators control the development of multicellular organisms by sequential activation. Zinc finger proteins, which possess zinc finger motifs in their core domain, comprise one such set of transcription factors. OVOL proteins are members of the zinc finger protein family and serve as transcription factors to regulate gene expression in various differentiation processes [1]–[3]. These genes function either as transcriptional activators or repressors [1], [4]–[7]. In *Drosophila melanogaster*, the OVO/Shavenbaby (Svb) is described as a complex gene with two genetic functions corresponding to separate control regions: OVO is required for female germline development and svb for epidermal morphogenesis [7]. *Drosophila* OVO/Svb is the best characterized among OVOL genes [8]. It consists of multiple spliced isoforms, which encode at least four different protein isoforms (A-D). All of these isoforms share four identical Cys2/His2 (C2H2) zinc fingers at their C-terminal ends, while they differ at the N-terminal portions.

An early germinal transcript of *Drosophila* encodes a transcriptional activator, OVOB, that is responsible for most of the OVO activity in females [1]. *Drosophila* OVO/Svb is a major regulator of epidermal differentiation: it triggers an early F-actin redistribution that initiates the cytoskeletal remodelling [9]. The N-terminal truncation of the OVO/Svb is mediated by four small 11–32 amino-acid-long polished rice (Pri) peptides (encoded by a small open reading frame, sORF) that convert it from a repressor to an activator [10]. Using this mechanism, Pri sORF peptides provide strict temporal control for the transcriptional program of epidermal morphogenesis during *Drosophila* embryogenesis [10]. A homolog of the OVO gene, lin-48 from *Caenorhabditis elegans,* encodes a C2H2 zinc-finger protein similar to the *Drosophila* OVO gene product [2]. Functional studies of OVOL genes and their involvement in epithelial differentiation have been reported for human and mouse OVOL genes such as OVOL1 and OVOL2. In addition, various functional studies in selected model organisms (e.g. flies, worms and mice) have further corroborated that OVOL genes are involved in the development and differentiation of epithelial cell lineages in a large spectrum of organisms [2], [7], [11]–[17].

To date, there are no studies on the molecular evolution of OVOL genes. This deficiency inspired us to investigate the molecular evolution of OVOL genes. Our approach aims to uncover the ancestors of OVOL genes and build a complete repository of OVOL genes from the currently available genomes of selected metazoans. We carried out a comparative genomic analysis of OVOL genes among metazoans focusing on the evolutionary processes involved in the origins of OVOL genes in vertebrates. We then further extended this analysis to early-branching metazoans. These analyses were based on protein sequences, structural features, phylogenies, micro-syntenies and the domain architecture of OVOL genes. From early-branching metazoans to humans, we found that a core domain of C2H2 zinc finger is conserved. The addition of various non-conserved sequences to this core, primarily to the N-terminal ends, gave rise to different types of OVOL genes during the course of metazoan evolution. Further, we report the orthologs and paralogs of different OVOL genes in selected metazoan genomes.

## Materials and Methods

### Extraction of Genomic, cDNA and Protein Sequences from Different Draft Genomes

We extracted the genomic DNA/cDNA/protein sequences from different eukaryotes via BLAST suite [18]–[20] using human/mouse OVOL1 as the query sequence against five genome databases: the National Centre for Biotechnology Information (NCBI) [21], the Department of Energy’s Joint Genome Institute (JGI; http://genome.jgi-psf.org/), the French National Sequencing Center Genoscope, (http://www.genoscope.cns.fr/externe/tetranew/), Ensembl [22], [23] and the *Strongylocentrotus purpuratus* genome at the Human Genome Sequencing Center (HGSC), Baylor College of Medicine (http://www.hgsc.bcm.tmc.edu/).We scanned the following vertebrates for putative OVOL genes: five teleosts: *Tetraodon nigroviridis* (*Tetraodon*) [24], *Takifugu rubripes* (*Takifugu*) [25], *Oryzias latipes* (medaka) [26], *Gasterosteus aculeatus* (stickleback) and *Danio rerio* (zebrafish)*;* one amphibian: *Xenopus tropicalis* (western clawed frog) [27] *;* three avian species: *Gallus gallus* (chicken) [28], *Taeniopygia guttata* (zebra finch) [29] and *Meleagris gallopavo* (turkey) [30]); one reptile: *Anolis carolinensis* (anole lizard) and four mammals: *Homo sapiens* (human) [31], *Mus musculus* (mouse) [32], *Rattus norvegicus* (rat) [33] and *Monodelphis domestica* (opossum) [34]. Further, we extended our analysis to different early-branching metazoan genomes such as *Branchiostoma floridae* (lancelets) [35], *S. purpuratus* (sea urchin) [36], *Trichoplax adhaerens* (placozoan) [37], *Nematostella vectensis* (sea anemone) [38], *Drosophila melanogaster* (fruit fly) [39], *C. elegans* (worm) [40], *Helobdella robusta* (annelid) and *Lottia gigantea* (mollusc).

### Synteny Analysis Using Selected Genomes

To unravel the orthologs and paralogs of human OVOL genes, we analyzed the synteny and orientation of conserved genes across different genomes using NCBI mapviewer [41] and the following genome browsers – ENSEMBL [22], [23], JGI, UCSC [42] and Genoscope (http://www.genoscope.cns.fr/externe/tetranew/).

### Sequence and Structural Analyses

We generated protein alignments of different OVOL proteins by MUSCLE [43], [44] using default parameters. We visualized raw muscle alignment using GENEDOC [45] without any column change from the output of MUSCLE [43], [44]. In this alignment, grey shades indicate 70% or higher conserved residues (with similar residues), while black shades show 100% conserved residues. We carried out secondary structure predictions of OVOL proteins using PSIPRED [46]. We predicted the ID regions in a given OVOL protein sequence using DISOPRED2 software [47]. DISOPRED2 is used to estimate the frequency of native disorder in several representative genomes from the three kingdoms of life [48]. This tool computes a position-specific scoring matrix (PSSM) and then analyses this matrix with an appropriately-trained support vector machine (SVM). Its accuracy has been shown to be ∼93.1% [49].

### Phylogenetic Analyses

We evaluated different amino acid substitution models using MEGA5 software suite [50], [51] and we found that the Dayhoff +G+I model [52] was the best fit to our dataset with the lowest Akaike Information Criterion (corrected AICc scores)  = 4054.1, with a proportion of invariable sites (I) = 0.217 and gamma (G) = 0.673. We inferred the evolutionary history of OVOL proteins from metazoan genomes by a Bayesian approach (5 runs, until average standard deviation of split frequencies was lower than 0.0098, 25% burn-in-period, Dayhoff +G+I matrix-based model method [52]) using the MrBayes 3.2 suite [53] with two alignments supplied in **supplementary Files S1** and **S2**.

### Distance Matrix Generation and Statistical Tests

We obtained p-distance matrices for OVOL aligned sequences using MEGA5 [50], [51]. Each pair-wise distance of any two different OVOL protein sequences within the alignment can be measured using this method, as previously shown [54]. To calculate the distances, we utilized the pair-wise deletion method. To estimate the variance, we used the bootstrap method. We also calculated the overall mean distance among all OVOL sequences for each data set. We imported the pair-wise distance values in the statistical package “R” [55] for statistical analysis and graphical representation. We generated box-plots to represent the degree of conservation using the statistical package “R” [55]. We carried out the Kruskal-Wallis analysis of variance test for each set of data to check the reliability and significance of the data points [56]. Pairwise diversity of OVOL protein sequences was measured for complete sequences, for conserved regions only, and for disordered regions only. In the graphical representation of the pairwise differences (values in the Y-axis), lower values indicate highly conserved sequences and higher values indicate more divergent sequences.

## Results and Discussion

### Protein Sequence and Structural Analysis of OVOL Proteins

**Figure 1:**
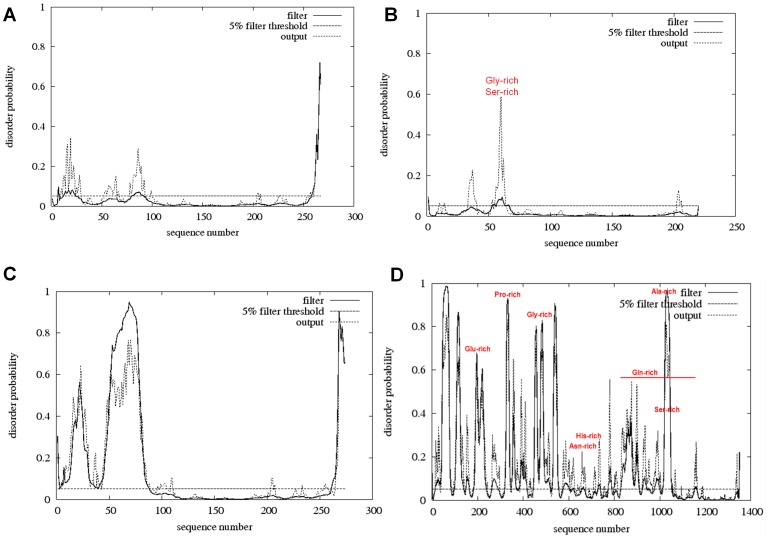
OVOL proteins are characterized by the presence of hypervariable ID regions. **A**. Mouse OVOL1 has ID residues in the first 100 amino acids. **B**. Mouse OVOL2 possesses ID residues in the first 50 amino acids with a glycine-rich and serine rich region as marked in red color. **C**. Mouse OVOL3 has ID segments within the N-terminal 100 residues. **D**. *Drosophila* OVO is intrinsically disordered with large patches of residue biasness as indicated by the red color. We used DISOPRED2 software [47] for the prediction of ID regions. The horizontal line indicates the ordered/disordered threshold for the default false positive rate of 5%. The 'filter' curve represents the outputs from DISOPRED2 and the 'output' curve represents the outputs from a linear support vector machine (SVM) classifier (DISOPREDsvm). The outputs from DISOPREDsvm are included to indicate shorter as low confidence predictions of disorder.

From the analysis of vertebrate OVOL protein sequences, we found that OVOL1, OVOL2, OVOL3 and OVOL3b share a common domain with a highly conserved tetrad of C2H2 type zinc finger motifs at the C-terminal region (**Supplementary figure S1**). Mammalian OVOL proteins have four C2H2-type zinc finger motifs in the following regions: 118–140 (23 aa long), 146–168 (23 aa long), 174–197 (24 aa long), and 213–236 (24 aa long) (numbering as per human OVOL1 protein sequence). Due to the N- and C-terminal extensions, the polypeptide length of OVOL proteins varies from 215–286 amino acids with OVOL1 being the longest and OVOL3 being the shortest polypeptides. The predicted secondary structure of human OVOL1 shows thirteen α-helices and eleven β-sheets as depicted in **supplementary figure S1**. There are small stretches of ID regions in the first 100 amino acids of the N-terminal regions of OVOL1-OVOL3 proteins (**Figures 1A–C**). The number of ID regions varies in different OVOL proteins and is lower in OVOL1 than in OVOL2-3. Hence, we can say that within ID proteins, structural disorder propensity may change between paralogs as previously reported [57].

**Figure 2:**
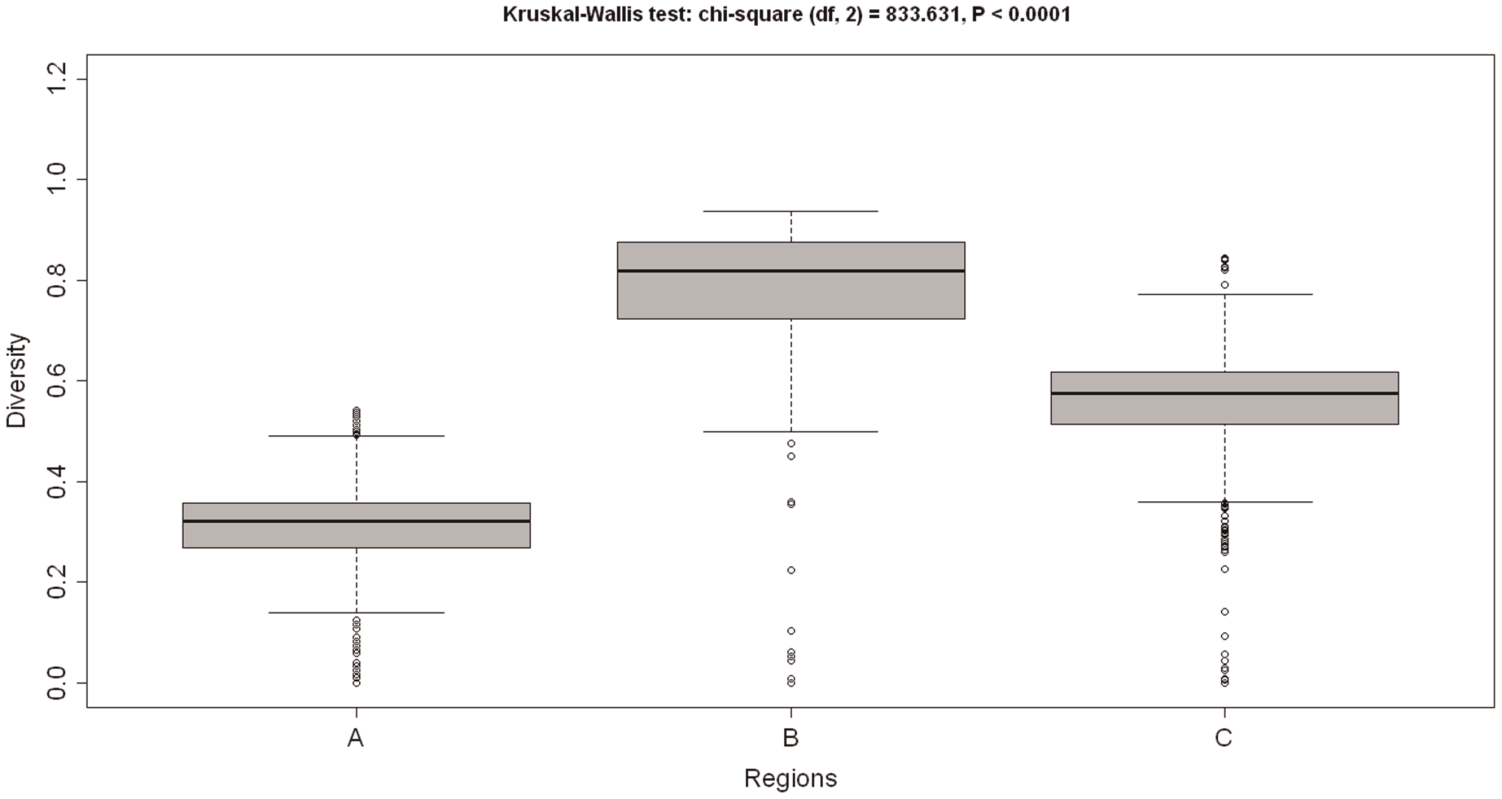
The disordered regions of OVOL have evolved more rapidly than structured regions. A) Structured regions only, B) Disordered segments only, C) Full-length OVOL.

Normally, *Drosophila* OVO is considered to be the ortholog of human and mouse OVOL genes. The *Drosophila* OVO gene encodes for four alternatively spliced isoforms named OVOA-D. *Drosophila* OVO-B isoform is the largest isoform, encoding a 1351 amino-acid-long polypeptide. Isoforms A, C and D are 975, 1222 and 1028 amino acids long, respectively. The *Drosophila* OVO has four C2H2 zinc finger motifs at the C-terminal end (**supplementary figure S2**) of almost the same length and located at the following positions: 1197–1219 (23 aa long), 1225–1247 (23 aa long), 1253–1276 (24 aa long), and 1292–1315 (24 aa long), respectively. Further, *Drosophila* OVO protein is also characterized by the presence of ID regions (**Figure 1D**), which are located at the following positions: a Glu-rich region from 196–239 (44 aa long), a Pro-rich region from 309–342 (34 aa long), a Gly-rich region from 448–618 (171 aa long), an Asn-rich region from 620–660 (41 aa long), a His-rich region from 645–665 (39 aa long), a Gln-rich region from 837–1158 (322 aa long), an Ala-rich region from 1001–1059 (59 aa long) and a Ser-rich region from 1025–1045 (21 aa long). The degree of disordered-ness as well as both the number and the positions of the ID regions are drastically different in *D. melanogaster* and in mammals. We found that the tetrad of C2H2 zinc finger motifs at the C-termini is highly conserved between *Drosophila* and mammals (**supplementary figure S2**). We found that ID regions rapidly evolved in comparison to the structured region of OVOL proteins as depicted in **Figure 2** **.** This concords with previous analyses by Brown *et al.* in which it has been shown that ID segments had higher rates of diversity than structured segments in a protein [58].

Furthermore, the simultaneous presence of secondary structural elements and disordered regions in vertebrate OVOL proteins is explained by overlaps of ordered and disordered regions and such zones are larger for small proteins [59]. Usually, ID proteins exhibit disorder-to-order transitions, which provide them the functional flexibility to adopt different structures while interacting with different partners [60]. The flexibility of ID proteins assists different disordered regions to bind to a common binding site on a common partner and such flexibility plays important roles in both protein–protein-interaction networks and gene-regulation networks [61]. Amino acid composition plays a decisive role in determining the ordered versus disordered status of a protein sequence with either highly order-promoting or highly disorder-promoting residues [60]–[63]. The extended regions in different OVOL proteins have no similarity and primarily consist of ID elements. These regions are frequently characterized by patches of a single type of residue occurring multiple times resulting in a non-foldable domain.

With several eukaryotic genomic sequences available, it is becoming more and more apparent that ID proteins are surprisingly common in eukaryotes and disordered domains are found in many functional proteins [64]–[67] including those involved in key regulatory processes such as cell signaling [68]–[70] and transcriptional regulation [71]. These ID segments have variable sizes ranging from a few to several hundred amino acids and sometimes constitute the entire protein, being as large as 200 kDa [72]. It is interesting that OVOL proteins with ID segments of such varied length are also transcriptional regulators. Generally, ID proteins or segments are non-homologous, being rapidly evolving and sequence-composition biased [72]. Thus, it is not surprising that the ID segments of OVOL proteins are non-homologous. These ID proteins are common in human diseases and thus this is often termed as disorder in disorders (D^2^) [60], [61]. However, their roles in disease are not known.

### Evolutionary History of OVOL Domains in Metazoans

We investigated the genomic locations and order of genes flanking OVOL genes to gain insight into the processes of OVOL gene evolution in vertebrates.

#### Genomic organization of OVOL1 orthologs in vertebrates

**Figure 3:**
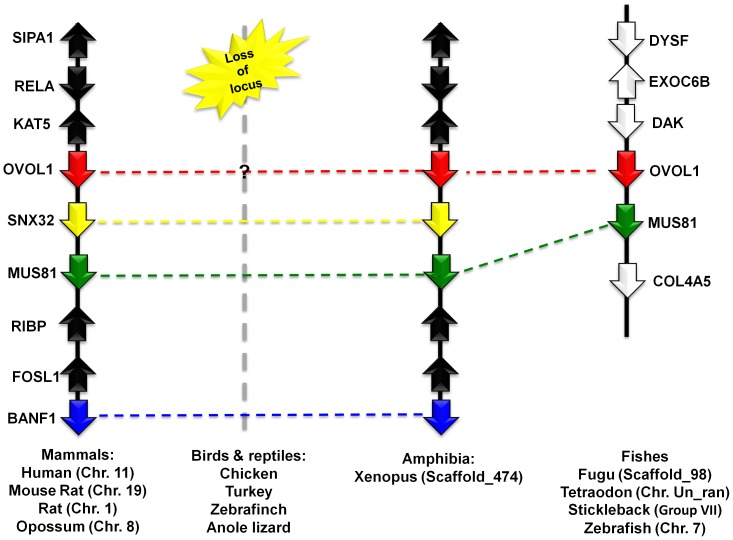
Chromosomal localization of OVOL1 gene from selected vertebrates, flanked by a set of conserved marker genes. SIPA1: signal-induced proliferation-associated 1; RELA: v-rel reticuloendotheliosis viral oncogene homolog A (avian); KAT5: K (lysine) acetyltransferase; SNX32: sorting nexin 32; MUS81: MUS81 endonuclease homolog (*S. cerevisiae*); BANF1: barrier to autointegration factor 1; EXOC6B: exocyst complex component 6B; DYSF: dysferlin, limb girdle muscular dystrophy 2B; COL4A5: collagen, type IV, alpha 5; DAK: dihydroxyacetone kinase 2 *S. cerevisiae* homolog.

The OVOL1 gene is localized on chromosome 11 in the human genome as shown in **Figure 3**. There is a conserved set of genes flanking the OVOL1 gene on both sides; a triad of SIPA1-RELA-KAT5 is present on one side and another set of five genes, namely SNX32-MUS81-RIBP-FOSL1-BANF1, is present on the other side. This set is confined in a region spanning about 380 kb. This syntenic architecture is maintained across several mammals including the mouse (chromosome 19/400 kb fragment), rat (chromosome 1/300 kb fragment), and opossum (chromosome 8/300 kb fragment). Interestingly, the entire genomic locus spanning the OVOL1 gene cluster in mammals is missing in the avian genomes (**Figure 3**) and in the anole lizard, *A. carolinensis* (AcoCar1.0 assembly), the only sequenced reptile genome. However, we traced OVOL1 in the amphibian *X. tropicalis* genome to a locus identical to that in mammals, in a 900 kb fragment on the scaffold_474. We also identified OVOL1 in different fish genomes, flanked by a triad of genes DYSF-ECOC6B-DAK on one side and by MUS81-COL4A5 on the other. This region spanned 300 kb, 340 kb, 350 kb and 380 kb in the *Takifugu* (scaffold_98), *Tetraodon* (chromosome Un_random), stickleback (groupVII) and zebrafish (chromosome 7), respectively. Although the sets of these flanking marker genes are different in tetrapods than in fishes, the presence of a single copy of a highly conserved gene – MUS81 encoding for a 611 amino-acid-long crossover junction endonuclease – is present in all vertebrate genomes. This strongly suggests that these OVOL1 loci are conserved only in fishes, amphibians and mammals. **Table 1** lists OVOL1 orthologs from different vertebrates.

**Table 1:** List of OVOL1 genes from selected vertebrate genomes, identified from Ensembl database release 58 (May 2010).

Organism	Scientific Name	Gene Id	Genomic Location	Transcript Id	Length (bp)	Protein Id	Length (aa)
**Human**	Homo sapiens	ENSG00000172818	Chromosome 11	ENST00000335987	2991	ENSP00000337862	267
**Mouse**	*Mus musculus*	ENSMUSG00000024922	Chromosome 19	ENSMUST00000025861	2900	ENSMUSP00000025861	267
**Rat**	*Rattus norvegicus*	ENSRNOG00000020669	Chromosome 1	ENSRNOT00000028081	2993	ENSRNOP00000028081	267
**Opossum**	*Monodelphis domestica*	ENSMODG00000009534	Chromosome 8	ENSMODT00000034545	810	ENSMODP00000032966	269
**Frog**	*Xenopus tropicalis*	ENSXETG00000020587	scaffold_474	ENSXETT00000044473	807	ENSXETP00000044473	268
**Fugu**	*Takifugu rubripes*	ENSTRUG00000011790	scaffold_98	ENSTRUT00000029879	961	ENSTRUP00000029762	286
**Stickleback**	*Gasterosteus aculeatus*	ENSGACG00000018794	groupVII	ENSGACT00000024893	1262	ENSGACP00000024844	274
**Tetraodon**	*Tetraodon nigroviridis*	ENSTNIG00000005499	Chromosome Un_random	ENSTNIT00000008360	846	ENSTNIP00000008194	281
**Zebrafish**	*Danio rerio*	ENSDARG00000079995	Chromosome 7	ENSDART00000113291	960	ENSDARP00000102900	256

#### Identification of OVOL2 orthologs and gene conservation in vertebrates

**Figure 4:**
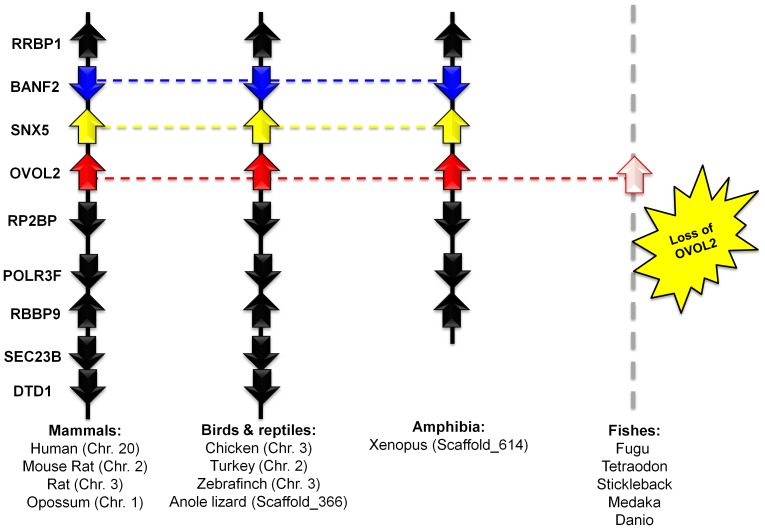
OVOL2 orthologs identified in vertebrates by comparing chromosomal localization. RRBP1: ribosome binding protein 1 homolog; BANF2: barrier to autointegration factor 2; SNX5: sorting nexin 5; CSRP2BP: CSRP2 binding protein; SEC23B: protein transport protein Sec23B; POLR3F: polymerase (RNA) III (DNA directed) polypeptide F; RBBP9: Retinoblastoma-binding protein 9; DTD1: D-tyrosyl-tRNA deacylase 1.

Upon tracing OVOL2 orthologs in different vertebrate genomes, we found that the OVOL2 gene is localized on chromosome 20 in the human genome flanked by a triad of RRBP1-BANF2-SNX5 on one side and a set of five genes, RP2BP-POLR3F-RBBP5-SEC23B-DTD1, on the other side in a region spanning about 900 kb (**Figure 4**). This genomic fragment is maintained in a wide variety of mammals including mice (chromosome 2/∼600 kb), rats (chromosome 3/600 kb fragment) and opossums (chromosome 1/1.3 Mb fragment). In the avian genomes, we detected similar fragments of 220 kb, 200 kb and 200 kb in chicken (chromosome 2), zebra finch (chromosome 3), and turkey (chromosome 2), respectively. Furthermore, we identified this syntenic organization in the anole lizard within a region of about 200 kb on scaffold_366. In contrast, we could not find OVOL2 in the fish genomes, even though we could detect the marker genes scattered on different loci. **Table 2** lists OVOL2 orthologs from different vertebrates.

**Table 2:** List of OVOL2 genes from selected vertebrate genomes, identified from Ensembl database release 58 (May 2010).

Organisms	Scientific Name	Gene ID	Chromosomal location	Transcript ID	Length (bp)	Protein ID	Length (aa)
**Human**	*Homo sapiens*	ENSG00000125850	Chromosome 20	ENST00000278780	1449	ENSP00000278780	275
**Mouse**	*Mus musculus*	ENSMUSG00000037279	Chromosome 2	ENSMUST00000037423	1539	ENSMUSP00000044026	274
**Rat**	*Rattus norvegicus*	ENSRNOG00000006850	Chromosome 3	ENSRNOT00000009226	1263	ENSRNOP00000009226	274
**Opossum**	*Monodelphis domestica*	ENSMODG00000005504	Chromosome 1	ENSMODT00000006948	798	ENSMODP00000006810	265
**Chicken**	*Gallus gallus*	ENSGALG00000008702	Chromosome 3	ENSGALT00000014161	816	ENSGALP00000014145	262
**Zebrafinch**	*Taeniopygia guttata*	ENSTGUG00000005909	Chromosome 3	ENSTGUT00000006128	789	ENSTGUP00000006069	263
**Turkey**	*Meleagris gallopavo*	ENSMGAG00000006770	Chromosome 2	ENSMGAT00000007574	504	ENSMGAP00000006816	167
**Anole Lizard**	*Anolis carolinensis*	ENSACAG00000014216	scaffold_366	ENSACAT00000014246	780	ENSACAP00000013960	260
**Frog**	*Xenopus tropicalis*	ENSXETG00000024897	scaffold_614	ENSXETT00000053523	2724	ENSXETP00000053523	287

#### Unraveling human OVOL3 orthologs in different vertebrates

**Figure 5:**
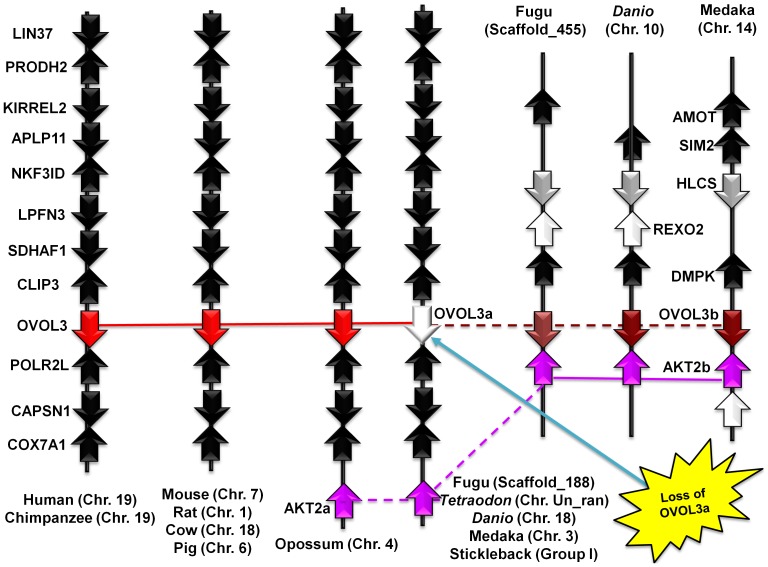
Synteny analysis of OVOL3 genes illustrates the loss of OVOL3a after duplication event and maintenance of paralogous OVOL3b in fishes. LIN37: lin-37 homolog (*C. elegans*); PRODH2: proline dehydrogenase (oxidase) 2; KIRREL2: kin of IRRE like 2 (*Drosophila*); APLP1: amyloid beta (A4) precursor-like protein 1; NFKBID: nuclear factor of kappa light polypeptide gene enhancer in B-cells inhibitor, delta; LRFN3: leucine rich repeat and fibronectin type III domain containing 3; SDHAF1: succinate dehydrogenase complex assembly factor 1; CLIP3: CAP-GLY domain containing linker protein 3; POLR2I: polymerase (RNA) II (DNA directed) polypeptide I, 14.5 kDa; CAPNS1: calpain, small subunit 1; COX7A1: cytochrome c oxidase subunit VIIa polypeptide 1 (muscle); DMPK: dystrophia myotonica-protein kinase; HLCS: holocarboxylase synthetase; AMOT: angiomotin; REXO2: REX2 RNA exonuclease 2 homolog (*S. cerevisiae*).

While tracing the OVOL genes, we identified a third OVOL gene, OVOL3, in a wide array of mammals including humans (chromosome 19), chimpanzees (chromosome 19), mice (chromosome 7), rats (chromosome 1), cows (chromosome 18), pigs (chromosome 6), and opossums (chromosome 4) with a conserved synteny. The conserved synteny comprises an octet of genes, LIN37-PRODH2-KIRREL2-APLP11-NKF3ID-LPFN3-SDHAF1-CLIF3, on one side and POLR2L-CAPSN1-COX7A1 on the other side of OVOL3 in a region of about 400 kb (**Figure 5**). Fishes lack the OVOL3 gene at this genomic arrangement; however, they possess another genomic organization that includes a similar OVOL gene, which we named OVOL3b. OVOL3b is flanked by a tetrad of genes formed by AMOT-HLCS-REXO2-DMPK on one side and by AKT2b on the other side on the scaffold_455 in *Takifugu.* A similar architecture is maintained in zebrafish (chromosome 10) and in medaka (chromosome 14). A complementary search for fish marker genes throughout the known mammalian genomes showed that AKT2 encodes for a kinase: RAC-beta serine/threonine-protein kinase. We found two copies of this gene in fishes: AKT2a and AKT2b. The AKT2a gene is found close to the cluster of genes that, in mammals, flank OVOL3, suggesting that OVOL3b in fishes is a paralog of OVOL3, developed by duplication of the OVOL3 locus. Subsequently, fishes lost OVOL3 in the original locus and the duplicated gene (OVOL3b) was retained at the duplicated locus. We found the cluster of genes that identify the duplicated OVOL3b gene at the duplicated loci in three fish genomes: fugu, zebrafish and in medaka. These fish genomes did not retain the original OVOL3 gene, offering rudimentary evidence of fish-specific whole-genome duplication events [73], [74] and subsequent loss of the original gene while a paralogous gene is maintained [75]. **Tables 3** and **4** list the OVOL3 orthologs from different vertebrates and paralogous OVOL3b genes of fishes, respectively.

**Table 3:** List of OVOL3 genes from selected mammalian genomes, identified from Ensembl database release 58 (May 2010).

Organisms	Scientific Name	Gene ID	Chromosomal location	Transcript ID	Length (bp)	Protein ID	Length (aa)
**Human**	*Homo sapiens*	ENSG00000105261	Chromosome 19	ENST00000262637	558	ENSP00000262637	185
**Chimpanzee**	*Pan troglodytes*	ENSPTRG00000010884	Chromosome 19	ENSPTRT00000020163	654	ENSPTRP00000018647	217
**Mouse**	*Mus musculus*	ENSMUSG00000056028	Chromosome 7	ENSMUST00000047308	821	ENSMUSP00000045372	220
**Rat**	Rattus norvegicus	ENSRNOG00000024880	Chromosome 1	ENSRNOT00000041301	615	ENSRNOP00000041919	205
**Cow**	Bos Taurus	ENSBTAG00000015001	Chromosome 18	ENSBTAG00000015001	666	ENSBTAP00000032029	222
**Pig**	Sus scrofa	ENSSSCG00000002924	Chromosome 19	ENSSSCT00000003230	636	ENSSSCP00000003149	212
**Opossum**	*Monodelphis domestica*	ENSMODG00000009534	Chromosome 8	ENSMODT00000034545	810	ENSMODP00000032966	269

**Table 4:** List of OVOL3b genes from fish genomes, identified from Ensembl database release 58 (May 2010).

Organism	Scientific Name	Gene Id	Genomic Location	Transcript Id	Length (bp)	Protein Id	Length (aa)
**Fugu**	*Takifugu rubripes*	ENSTRUG00000008625	scaffold_455	ENSTRUT00000021725	393*	ENSTRUP00000021637	131
**Medaka**	*Oryzias latipes*	ENSORLG00000005380	Chromosome 14	ENSORLT00000006784	654	ENSORLP00000006783	218
**Zebrafish**	*Danio rerio*	ENSDARG00000079995	Chromosome 10	ENSDART00000108918	960	ENSDARP00000099960	253

* Partial sequences.

On close inspection of the syntenic arrangements of OVOL1-OVOL3 genes in vertebrates, we found that syntenies of OVOL1 and OVOL2 share marker genes that are homologous as well. For example, Barrier-to-autointegration factor homologs BANF1 and BANF2 (marked by blue color in **Figures 3** **and** **4**), flanked OVOL1 and OVOL2 respectively. The same is true for sorting nexin homologs, SNX32 and SNX5 (marked by yellow color in **Figures 3** **and** **4**), which again flank OVOL1 and OVOL2 respectively. These data indicate that OVOL1 and OVOL2 originated by fragmental duplications more than 450 MY ago, as the homologous gene cluster is maintained from fish to mammals. Surprisingly, birds have only one copy of OVOL genes. In summary, we have developed a catalogue of OVOL genes from vertebrates, which originated from duplication events prior to the separation of fishes from the tetrapod lineage about 450 MY ago.

**Figure 6:**
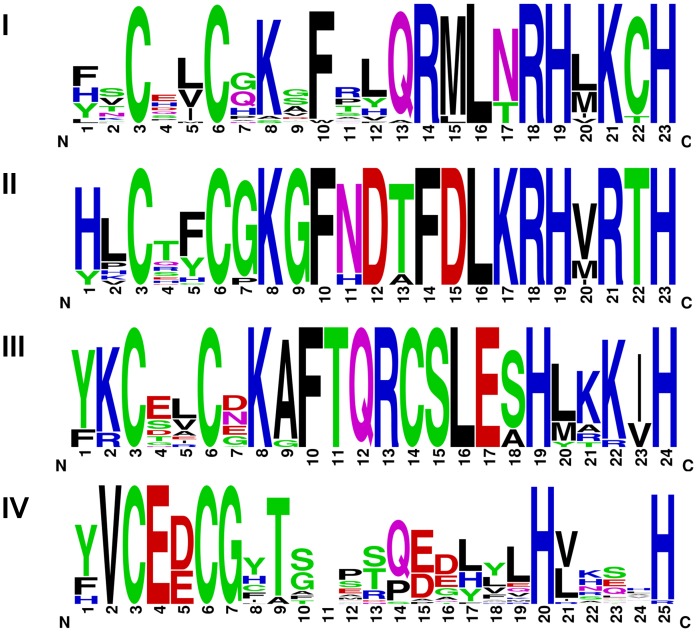
Sequence logo of four different Cys2-His2 (C2H2) zinc finger motifs (I-IV) present in different OVOL proteins from metazoan genomes. We generated this sequence logo using WebLogo 3.0 [81]. C2H2 zinc finger motif IV has 25 amino acids due to the presence of one extra amino acid at the eleventh position in the OVOLNVE1 protein from sea anemone.

To further characterize the OVOL proteins among different metazoans, we traced OVOL genes in a set of basal metazoan genomes. Based on similarity searches for human OVOL genes, we identified two genes from the *B. floridae* genome (JGI accession id e_gw.374.48.1 and e_gw.236.92.1) that share a high degree of conservation. We identified two genes in the sea anemone (*N. vectensis*) genome (JGI accession id gw.31.97.1 and e_gw.31.122.1), which have highly conserved OVO domains compared to humans. In these two species, OVOL proteins show a single domain with a tetrad of C2H2 zinc finger motifs. Further searches with human OVOL proteins as query, allowed us to identify two OVOL proteins in the leech (*H. robusta*; annelid) genome (accession id e_gw1.1.1891.1 and e_gw1.4.1162.1) and two OVOL genes in the genome of *L. gigantea* (JGI accession ids fgenesh2_pg.C_sca_13000299 and e_gw1.13.34.1). Two OVOL proteins (accession id XP_796652.2 and XP_788176.1) were detected in the sea urchin (*S. purpuratus*) genome. These two *S. purpuratus* proteins share a conserved C2H2 zinc finger domain in addition to non-homologous N-terminal extensions. Finally, we identified one OVOL gene in the placozoan *T. adhaerens* genome (accession id e_gw1.4.509.1). The comparisons of the four C2H2-type zinc finger motifs show that they are highly conserved from early-branching eukaryotes to vertebrates **(** **Figure 6** **)**. These are typical C2H2 type zinc finger motifs, as described in the Prosite pattern database (accession number PS00028) [76], with a few exceptions. OVOL C2H2 motif IV is highly divergent among these motifs while C2H2 motifs II and III are highly conserved as shown by pattern comparisons (**Table 5**). Interestingly, all C2H2 motifs have a conserved C-x(2)-C-x(3) pattern and a phenylalanine (F) is found next to it in a majority of cases, with some exceptional residues in C2H2 motif IV. The overall C2H2 pattern for OVOL is C-x(2)-C-x(3)-[FWsgat]-x(8)-H-x(3,4)-H, which is a subset of the C2H2 motif described by Prosite (with exceptional residues marked in lower case).

**Table 5:** Pattern comparisons for four C2H2 motifs from OVOL proteins. Exceptional residues are marked by lower case.

Typical C2H2 (PS00028) motif		C-x(2,4)-C-x(3)-[LIVMFYWC]-x(8)-H-x(3,5)-H
**OVOL C2H2**	**I**	C-x(2)-C-x(3)-[FW]-x(8)-H-x(3)-H
	**II**	C-x(2)-C-x(3)-[F]-x(8)-H-x(3)-H
	**III**	C-x(2)-C-x(3)-[F]-x(8)-H-x(4)-H
	**IV**	C-x(2)-C-x(3)-[Fsgat]-x(8)-H-x(4)-H
**Overall OVOL C2H2 pattern**		C-x(2)-C-x(3)-[FWsgat]-x(8)-H-x(3,4)-H

x(3) - any residues at next three positions,

x(2,4) – any residue at 2–4 variable positions,

[LIVMFYWC] – one of the listed residues at this position.

We further detected a new motif present in the majority of OVOL proteins from different metazoans. This motif is present in the C-terminal end immediately after the fourth C2H2 motif and it corresponds to the 237–242 and 1316–1321 positions in human OVOL1 and *Drosophila* OVOB (**supplementary figure S3**), respectively. By using various motif-scanning strategies to different databases, we can suggest that this is a novel motif, which is not found in any other transcription factors known so far. At this point, we are unable to assign any functional role to this novel motif.

**Figure 7:**
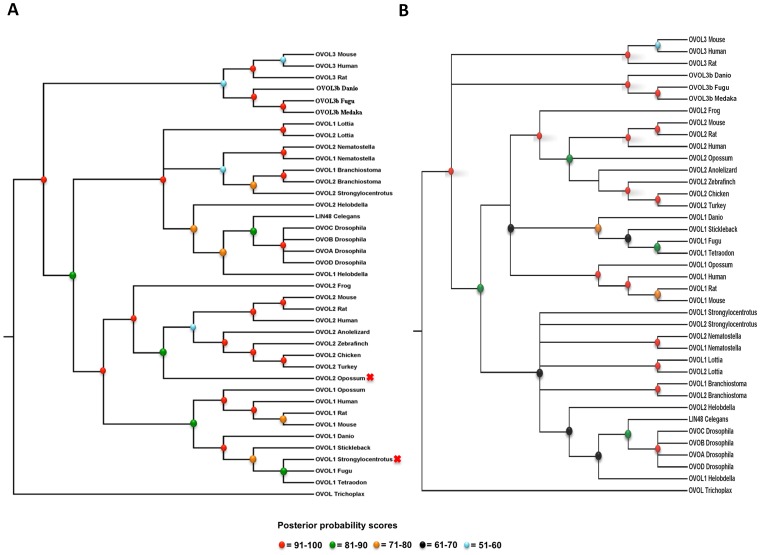
Phylogenetic history of OVOL proteins using the Bayesian method. A. Full-length OVOL proteins. B. Selected region of OVOL proteins. Posterior probabilities scores are depicted by various color balls. The placozoan OVOL protein (e_gw1.4.509.1) was used as the outgroup in this phylogenetic tree. Red x indicates sequence position, which did not accord with species phylogeny. BFL: *B. floridae* (lancelet), SPU: *S. purpuratus* (sea urchin), NVE: *N. vectensis* (sea anemone), HRO: *H. robusta* (annelids), LGI: *L. gigantean* (molluscs) and TAD: *T. adhaerens* (placozoan). Trees in figures 7A and 7B are generated using the MrBayes 3.2 [53] from alignments supplied in supplementary Files S1 and S2, respectively.

Strikingly, in every animal genome (either completely sequenced or available as a draft), we found at least one OVOL gene, corroborating the hoariness of these genes and their strong conservation during metazoan evolution. To infer the phylogenetic relationships among OVOL proteins from early-branching eukaryotes to vertebrates, we reconstructed a Bayesian phylogenetic tree (**Figure 7**). We used OVOL protein from placozoan as an outgroup. The OVOL proteins branched in a lineage-specific manner with a few exceptions marked by a red “x” in **figure 7A**. However, by deleting non-homologous segments from the protein alignment (supplemented in **Supplementary File S2**) OVOL proteins clustered according to the expected taxonomic relationships in the phylogenetic tree (**Figure 7B**).

Vertebrate OVOL1 and OVOL2 branch out together, which corroborates their common ancestry as supported by their syntenic analyses (described above). The protozoan OVOL gene appears to be the sister clade to the three OVOL genes present in vertebrates. This is probably due to the fact that it is composed of a highly conserved C2H2 zinc finger domain. This C2H2 domain is strongly conserved, while ID regions have no homologous segments across metazoa.

**Figure 8:**
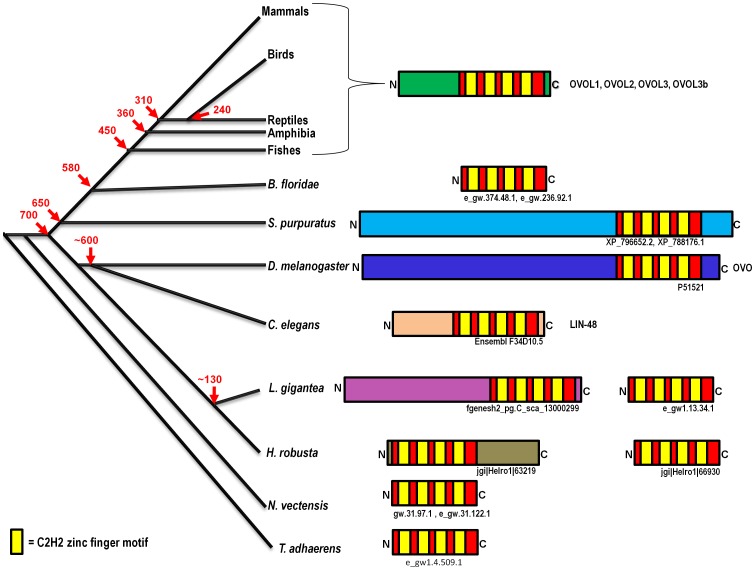
Protein domain evolution of OVOL proteins from different metazoan lineages over a period of >700 MY. A highly conserved domain of a tetrad of C2H2 zinc finger motifs (red and yellow box) is found in various metazoa. Primarily, the N-terminal extensions in C2H2 lead to different types of protein with the exceptions of OVOL proteins from the leech and sea urchin where extension was found in the C-terminal end of C2H2 zinc finger motif. The ID segments with no homology in evolutionary distant organisms are marked in different colors. Times of divergence are taken from Kumar and Hedge (2003) [82] and Ponting (2008) [83].

The evolution of OVOL protein domains spans a period of more than 700 million years from placozoans to humans, shown in **Figure 8**. Basal metazoans, such as the sea anemone, possess only a tetrad C2H2 zinc-finger-carrying OVOL domain, to which different types of OVOL proteins are built by different N−/C-terminal extensions. These extensions are predominantly N-terminal with some exceptions in annelids and sea urchins. The extension of these peptides ranges from one hundred to several hundreds of amino acids. For example, vertebrate OVOL proteins and LIN48 from *C. elegans* have about 100–120 amino acid extensions with ID regions. In contrast, both *Drosophila* and *S. purpuratus* OVOL proteins have extensions of several hundreds of amino acids at the N-terminal end. The extended amino acid regions are not homologous with other OVOL proteins from evolutionarily distant organisms. It is expected that full-length domains of an ortholog will be conserved. Therefore, our findings indicate that these proteins from different lineages are in fact “homologs”, but not necessarily orthologs, of *Drosophila* OVO proteins as has been described in annotations across different databases such as the Ensembl release 58 [22], [23]. Orthologous proteins share a conserved core domain with comparable lengths of polypeptides, which is not valid in case of OVOL proteins. Hence, this poses a notorious case for orthology assignment. Genes are usually described as co-orthologous [77] in such complex cases.

It is clear from our study that the ID regions of OVOL proteins do not share homologous regions beyond their own lineage. These ID regions can maintain cryptic genetic variability [78] and explain the *de novo* creation of genes, such as can be seen in the 60 *de novo* genes in the human genome [79], [80]. This corroborates that it is possible that a segment of gene can also be originated by a *de novo* mechanism. In the case of OVOL, it can be explained by the following steps: (i) a non-coding DNA acquires properties of coding DNA and most likely becomes a transcribable and translatable exon, (ii) fusion of this *de novo* coding region or exon with a highly-conserved pre-existing C2H2 zinc-finger gene. We cannot ignore this possibility, but further work is needed to find the exact mechanism.

### Conclusions

To the best of our knowledge, this is the first comprehensive and systematic study that explains the molecular evolution of OVOL genes. We found that the OVOL proteins are composed of a single zinc finger domain (with a highly conserved tetrad of C2H2), which has been conserved from eumetazoans to humans. By N−/C-terminal expansion to this conserved domain, these proteins rapidly acquired extra segments that are primarily comprised of ID regions without significant sequence similarities. These ID regions have originated independently across different animal lineages. This study significantly advances our understanding of the evolution of OVOL genes in metazoa and provides a platform for further characterization as more metazoan genomes are expected to be available soon.

## Supporting Information

Figure S1 **Alignment of OVOL proteins from different vertebrates,** ***B. floridae and N. vectensis.*** We created this alignment using MUSCLE [43], [44] and further edited for visualization using GENEDOC [45]. Secondary structures of human OVOL1 were predicted using PSIPRED [46] and these secondary structures are marked above the alignment. Four C2H2 zinc finger motifs (I-IV) are marked by the orange bar. The rodent OVOL3 protein terminates at position 10 in C2H2 motif IV. Grey and back shades indicate 70% and over conserved residues (with similar residues) and 100% conserved residues, respectively.
(PDF)

Figure S2 **Similarities and differences among** ***Drosophila*** **OVOA-D and mouse OVOL1-OVOL3 using protein sequence alignment.** Zinc finger motif is a highly conserved region (red shading). The presence of multiple stretches of the same amino acids are visible in this alignment in the N-terminal regions.
(PDF)

Figure S3 **A highly conserved motif is found immediately after the fourth C2H2 motif present in the majority of OVOL proteins from metazoan origin.** We generated this sequence logo using WebLogo 3.0 [81].
(PNG)

File S1 **Protein sequence alignment of full length OVOL.** We generated this alignment using MUSCLE [43], [44] at default parameters. This alignment was utilized for the reconstruction of the Bayesian phylogenetic tree (**Figure 7A** **)**.
(TXT)

File S2 **Protein sequence alignment of selected regions of OVOL.** We generated this alignment using MUSCLE [43], [44] at default parameters. This alignment was used for the reconstruction of the Bayesian phylogenetic tree (**Figure 7B** **)**.
(TXT)
