# Supplementary material for: Molecular Phylogeny of OVOL Genes Illustrates a Conserved C2H2 Zinc Finger Domain Coupled by Hypervariable Unstructured Regions
Source: PLoS One. 2012 Jun 21;7(6):e39399. doi: 10.1371/journal.pone.0039399 (PMC3380836; doi:10.1371/journal.pone.0039399)
Supplement: Figure S1 — Alignment of OVOL proteins from different vertebrates, B. floridae and N. vectensis. We created this alignment using MUSCLE [43], [44] and further edited for visualization using GENEDOC [45]. Secondary structures of human OVOL1 were predicted using PSIPRED [46] and these secondary structures are marked above the alignment. Four C2H2 zinc finger motifs (I-IV) are marked by the orange bar. The rodent OVOL3 protein terminates at position 10 in C2H2 motif IV. Grey and back shades indicate 70% and over conserved residues (with similar residues) and 100% conserved residues, respectively. (PDF) [file pone.0039399.s001.pdf]

## Figure S1

```
                                β1        β2  α1            α2   β3
OVOL1-Human          1   MPRAFLVKKPCVSTCKRNWSELPDEERGEIYVP--VSLGFCPP-------QPYREP   47
OVOL1-Mouse          1   MPRAFLVKKPCVSTCKRNWSELPDEERGEIYVP--VSLGFCPP-------QPYREP   47
OVOL1-Rat            1   MPRAFLVKKPCVSTCKRNWSELPDEERGEIYVP--VSLGFCPP-------QPYLEP   47
OVOL1-Opossum        1   MPRAFLVKKPCVSTGKRNWSELPDEERGEIYLP--ARLSGLLSG------DPLEGG   48
OVOL1-Stickleback    1   MPRTFLVKKSNVSPGKRNWSEVPDHERGDVYVP--VPLTFC---------DVKQQL   45
OVOL1-Fugu           1   MPRAFLVKKANVSPGKRNWSEVSDHERGDVYIPDHVGISCFLKNKQ----PPQASK   52
OVOL1-Tetraodon      1   MPRAFLVKKANVSPGKRNWSEVPDHERGDVYIP--ASTPVEADCLRK---VHSRCC   51
OVOL1-Danio          1   -----------MYTHSQMYTNDPGAVVPVAELP--SCTALGQS-------PETEHI   36
OVOL2-Human          1   MPKVFLVKRRSLGVSVRSWDELPDEKRADTYIP--VGLGRLLHDP-----PEDCRS   49
OVOL2-Mouse          1   MPKVFLVKRRSPGVSVRSWDELPDDKRADTYIP--VSLGCLLRDP-----PEDCRS   49
OVOL2-Rat            1   MPKVFLVKRRSPGASVRSWDELPDDKRADTYIP--VSLGCLLRDP-----PEDCRS   49
OVOL2-Opossum        1   MPKVFLVKRRNPVATIRSWDELPDEERADTYIP--VGWGVGKSRP-----RIKCVC   49
OVOL2-Chicken        1   MPRAFLVKRRSPQPALRSWAGLPDDERADTYIP--SGLGCAPGY------EDSCSL   48
OVOL2-Zebrafinch     1   MPRAFLVKRRSPQPAVRSWDGLPDEERADTYIP--GGIGCVLLGY-----EDSCSL   49
OVOL2-Anolelizard    1   MPRAFLVKRRNPLPASRSWDALPDEERADTYVP--APFPLVLHR------PDYCKK   48
OVOL2-Frog           1   MPRAFLVKRRSPPPVIRNWDELPDEERADTYIP--DTIDCIFKYVTEDSLSNSSAG   54
OVOL3-Human          1   MPRAFLVRSRRPQPP--NWGHLPDQLRGDAYIP--GG-------------------   33
OVOL3-Mouse          1   MPRVFLVRSRRPQPP--NWSHLPDQLRGDAYVP--GSWLPLLH-------PGPSST   45
OVOL3-Rat            1   MPRVFLVRSRRPQPP--NWSHLPDQLRGDAYVP--G--------------MHLSGP   38
OVOL3b-Danio         1   MPRSFLVKRRQAAGAGWTW---KDTETTHTHTA--AA-------------HTHTSL   38
OVOL3b-Fugu          -   --------------------------------------------------------   -
OVOL3b-Medaka        1   MPRSFLVKKKRGACGAWQWKE-PDKTIV----------------------SEHPTK   33
OVOLBFL1             -   --------------------------------------------------------   -
OVOLBFL2             -   --------------------------------------------------------   -
OVOLNVE2             1   ---------------------MKDEQ------------------------------   5
OVOLNVE1             -   --------------------------------------------------------   -

                              α3         α4                                α5
OVOL1-Human         48   EPSVAEPPSCPLALNMSLRDSSYSMAPGPCVVAQLPS-------EDMGHLTDPQSR   96
OVOL1-Mouse         48   EASVAEPPSCPLALDMSLRDSSYSVAPGPCVVAQLPS-------EDVSHLTDPQSR   96
OVOL1-Rat           48   EASVAEPPSCPLALDMSLRDSSYGVASGPCEVAQLPP-------EDVNHLTDPQSR   96
OVOL1-Opossum       49   GGAVALGGSPSERRGKGGAAWGTGRVLLPASRLGHPS-------DTLAPDTDTPTR   97
OVOL1-Stickleback   46   HFCNVNIFPPPPQITTASLMPHATEMPVALTSTPEPV-------EAVSMVTRSTGQ   94
OVOL1-Fugu          53   TMSEHHAGTSAEPSHSARGNVRDEEEAAPGEPDLHPI------QNESEHAPASKPS  102
OVOL1-Tetraodon     52   KSVWSDELNCITDVVTTGELPAVTTPSSPTPISKAPA-------KNQHVPASKTSS  100
OVOL1-Danio         37   RSTQNNTYVRTKIKVTTGELPTEAPPVVPSVTTSPPV--------SIATHCSPPVT   84
OVOL2-Human         50   DGGSSSGSGSSSAGEPGG-AESSSSPHAPESETPEPG-------DAEGPDGHLATK   97
OVOL2-Mouse         50   DGGSSSG--CSSSAGEPGGAESSSSPRAPEPETPELH-------DAQGTDGHLAAM   96
OVOL2-Rat           50   DGGSSSGC-SCSAGEPGG-AESSSSPRAPEPETPELH-------DAEGTDGHLAAM   96
OVOL2-Opossum       50   GGGSSSGGSVQESEPR--------DHLAPQSGAPEPR--------TNEEPVLHLAG   89
OVOL2-Chicken       49   ESSGSSGTRDPELGDP----------PTPQAAPAELG--------APGGMLLDLAV   86
OVOL2-Zebrafinch    50   ESSGSSGTRDAEPSDP----------PTPQPAPGELG--------TGGGMLLDLAV   87
OVOL2-Anolelizard   49   QGKPSEGNPNSKAQPQNSCSLESTSSRDTETANISAE-------STTEKTLLALAE   97
OVOL2-Frog          55   DPAGDSPERSSSDSGSCNGSITTPPHNTPQSGLVSPSRKDSTKQEDEEEQTSDPHG  110
OVOL3-Human         34   -PLTVPGGKGQERRSVTI---------------WLFS-------SDCSSLGGPPAQ   66
OVOL3-Mouse         46   QSQGSVFSGCEEDSKKGGGSVSLTSQDCPSLWAHNYR--------VAKTEQDPAGH   93
OVOL3-Rat           39   GGEGLGGTGYHHLASL-------PRLQQPGSATSTQL-------FWSERHVGSVSV   80
OVOL3b-Danio        39   DSVCAPAVQKHTAAPAAAALERHRGDYVMERLPPPPP-------AALSSSAAALHK   87
OVOL3b-Fugu          -   --------------------------------------------------------   -
OVOL3b-Medaka       34   EASFAMETRLPPTVHPTVGMSAHGAAEAPVPVPFTGP-------GDDGRQKWSKPR   82
OVOLBFL1             -   --------------------------------------------------------   -
OVOLBFL2             -   --------------------------------------------------------   -
OVOLNVE2             -   --------------------------------------------------------   -
OVOLNVE1             -   --------------------------------------------------------   -
```

```
                                         C2H2-I

                    β4    α6          α7    β5      β6
OVOL1-Human         97  DHGFLRTKM-KVTLGDSPSG-DLFTCRVCQKAFTYQRMLNRHMKCHNDVKRHLCTY  150
OVOL1-Mouse         97  DQGFLRTKM-KVTLGDSPNG-DLFTCHICQKSFTHQRMLNRHMKCHNDVKRHLCTY  150
OVOL1-Rat           97  DQGFLRTKM-KVTLGDSPNG-DLFTCHICQKSFTYQRMLNRHMKCHNDVKRHLCTY  150
OVOL1-Opossum       98  PRALSRSKLGRVTMGDSPSG-DLFTCHICHKAFTYQRMLNRHMKCHNDVKRHLCTY  152
OVOL1-Stickleback   95  SQ--------SLLTGMKGPT-GAYVCHVCQKTFSYQRMLNRHLKCHNDTKRHLCSF  141
OVOL1-Fugu         103  SAPVGATMLTRSTAQNQNLSAVTFVCQVCQKVFLHQRMLNRHVKCHSDTKRHLCSF  158
OVOL1-Tetraodon    101  AAPDAAMVT-RSTAQNQNSP---FVCQVCQKAFHHQRMLNRHLKCHSETKRHLCSF  152
OVOL1-Danio         85  KPLMVQSSG------------ATYVCQVCQKVFQFQRMLNRHLKCHSEQKRHLCDF  128
OVOL2-Human         98  QRPVARSKI-KFTTGTCSDS-VVHSCDLCGKGFRLQRMLNRHLKCHNQVKRHLCTF  151
OVOL2-Mouse         97  QRPVARSKI-KFTTGTCDNS-VIHNCDLCGKSFRLQRMLNRHLKCHNQVKRHLCTF  150
OVOL2-Rat           97  QRPVARSKI-KFTTGTCNDS-VIHNCDLCGKSFRLQRMLNRHLKCHNQVKRHLCTF  150
OVOL2-Opossum       90  KRPTARSKI-KFTTGTCNEA-MVYSCELCGKGFRLQRMLNRHLKCHNQVKRHLCTF  143
OVOL2-Chicken       87  RRPAVRSKI-KFTTGTCTDA-AVHSCELCGKGFRLQRMLNRHVKCHSQVKRHLCTF  140
OVOL2-Zebrafinch    88  KRPMVRSKI-KFTTGTCNDA-ALHSCELCGKGFRLQRMLNRHIKCHSQVKRHLCTF  141
OVOL2-Anolelizard   98  NRPTVRSKI-KFTTGICGDA-LMHNCELCGKGFRLQRMLNRHIKCHSQVKRHLCTF  151
OVOL2-Frog         111  KRPSARSKI-KFTTGSCSE--EIYSCEICAKGFRLQRMLNRHLKCHNQVKRHLCTF  163
OVOL3-Human         67  QSSSVRDP------------------------WT-ARMLTRHLKCHSPVRRHLCRC   97
OVOL3-Mouse         94  TNPYTLWQSSQGTLTSAPKGPGTLGCPLCPKAFPLQRMLTRHLKCHSPARRHVCHY  149
OVOL3-Rat           81  RTPIPRSSQ-GTLTSVPKGP-GTLSCPLCPKAFPLQRMLTRHLKCHSPARRHVCHY  134
OVOL3b-Danio        88  PRDASSAGS------------SEFLCSVCHKLFPLQRMLTRHLKCHSLIKRHPCRY  131
OVOL3b-Fugu          1  -------------------------CSMCHKIFPLQRMLTRHLKCHSLVKRHPCQF   31
OVOL3b-Medaka       83  PRPSTVS--------------GDFVCSVCHKVFPLQRMLTRHLKCHSLVKRHPCRF  124
OVOLBFL1             1  ---------------------------LCGKDFAVQRMLNRHMKCHSATKRYKCEH   29
OVOLBFL2             1  ---------------------------LCGKDFAVQRMLNRHMKCHSATKRYKCEH   29
OVOLNVE2             6  ---------------------NKYKCEICQSSFSLQRLLNRHMKTHSFYKRYHCQF   40
OVOLNVE1             1  -----------------------YKCGLCNASFSLQRLLNRHMKTHSFYKRYHCQF   33
```

```
                          C2H2-II                  C2H2-III

                       α8      β7    α9        β8   α10     β9        α11
OVOL1-Human        151  CGKGFNDTFDLKRHVRTHTGVRPYKCSLCDKAFTQRCSLESHLKKIHGVQQKYAYK  206
OVOL1-Mouse        151  CGKGFNDTFDLKRHVRTHTGVRPYKCSLCDKAFTQRCSLESHLKKIHGVQQKYAYK  206
OVOL1-Rat          151  CGKGFNDTFDLKRHVRTHTGVRPYKCSLCDKAFTQRCSLESHLKKIHGVQQKYAYK  206
OVOL1-Opossum      153  CGKGFNDTFDLKRHVRTHTGVRPYKCSLCDKAFTQRCSLESHLKKIHGVQQKYAYK  208
OVOL1-Stickleback  142  CGKGFNDTFDLKRHVRTHTGVRPYKCTLCDKAFTQRCSLESHMKKIHSVTQKYAYK  197
OVOL1-Fugu         159  CGKGFNDTFDLKRHVRTHTGVRPYKCTLCEKAFTQRCSLESHMKKIHSITQAYAYK  214
OVOL1-Tetraodon    153  CGKGFNDTFDLKRHVRTHTGVRPYKCTLCEKAFTQRCSLESHMKKIHSITQTYAYK  208
OVOL1-Danio        129  CGKGFNDTFDLKRHVRTHTGVRPYKCELCDKAFTQRCSLESHMKKIHGVTQQYAYK  184
OVOL2-Human        152  CGKGFNDTFDLKRHVRTHTGIRPYKCNVCNKAFTQRCSLESHLKKIHGVQQQYAYK  207
OVOL2-Mouse        151  CGKGFNDTFDLKRHVRTHTGIRPYKCEVCNKAFTQRCSLESHLKKIHGVQQQYAYK  206
OVOL2-Rat          151  CGKGFNDTFDLKRHVRTHTGIRPYKCEVCYKAFTQRCSLESHLKKIHGVQQQYAYK  206
OVOL2-Opossum      144  CGKGFNDTFDLKRHVRTHTGIRPYKCEICNKAFTQRCSLESHLKKIHGVQQQYAYK  199
OVOL2-Chicken      141  CGKGFNDTFDLKRHVRTHTGIRPYKCEVCNKAFTQRCSLESHLKKIHGVQQQYAYK  196
OVOL2-Zebrafinch   142  CGKGFNDTFDLKRHVRTHTGIRPYKCEVCNKAFTQRCSLESHLKKIHGVQQQYAYK  197
OVOL2-Anolelizard  152  CGKGFNDTFDLKRHVRTHTGIRPYKCEVCNKAFTQRCSLESHLKKIHAVQQQYSYK  207
OVOL2-Frog         164  CGKGFNDTFDLKRHVRTHTGIRPYKCEICNKAFTQRCSLESHLKKIHGVQQGYAYK  219
OVOL3-Human         98  CGKGFHDAFDLKRHMRTHTGIRPFRCSACGKAFTQRCSLEAHLAKVHGQPASYAYR  153
OVOL3-Mouse        150  CGKGFHDAFDLKRHMRTHTGIRPFRCGACGKAFTQRCSLEAHLAKVHGQPASYAYR  205
OVOL3-Rat          135  CGKGFHDAFDLKRHMRTHTGIRPFRCGACGKAFTQRCSLEAHLAKVHGQPASYAYR  190
OVOL3b-Danio       132  CGKGFNDTFDLKRHMRTHTGIRPYRCDLCEKAFTQRCSLESHLRKIHGVQQQYAYR  187
OVOL3b-Fugu         32  CGKGFNDTFDLKRHMRTHTGIRPYKCELCEKAFTQRCSLESHMRKIHGVHQQYAYR   87
OVOL3b-Medaka      125  CGKGFNDTFDLKRHMRTHTGIRPYKCELCEKAFTQRCSLESHMRKIHGVHQQYAYR  180
OVOLBFL1            30  CPKGFNDTFDLKRHVRTHTGIRPYKCEECGKGFTQRCSLESHYKKVHGLTLSYGYK   85
OVOLBFL2            30  CPKGFNDTFDLKRHVRTHTGIRPYKCEECGKGFTQRCSLESHYKKVHGLTLSYGYK   85
OVOLNVE2            41  CGKGFNDTFDLKRHIRTHTGIKPFKCDRCDKAFTQRCSLEAHLTRVHSVVHKYGFR   96
OVOLNVE1            34  CGKGFNDTFDLKRHIRTHTGIKPFKCDRCDKAFTQRCSLEAHLTRVHSVVHKYGFR   89
```

C2H2-IV

```
                                α12       α13        β10
OVOL1-Human        207  ERRAKLYVCEECGCTS-ESQEGHVLHLKEHHPDSPLLR-KTSKKVA-----VALQN  255
OVOL1-Mouse        207  ERRAKLYVCEECGCTS-ESQEGHVLHLKERHPDSPLLR-KTSKKVA-----VALQN  255
OVOL1-Rat          207  ERRAKLYVCEECGCTS-ESQEGHVLHLKEHHPDSPLLR-KTSKKVA-----VALQN  255
OVOL1-Opossum      209  ERRAKLYVCEECGCTS-DSQEGHVLHLKEHHPDSPLLR-KTSKKGA-----GALQN  257
OVOL1-Stickleback  198  ERRNKLYVCEECGLTA-ATQDALLLHLHSLHPDSALLKGKAARRAAGGGGGGGAEG  252
OVOL1-Fugu         215  ERRNKLYVCEECGHTS-GSQDDLLVHLHLLHPNSHLLKGKTARRTG--GGKPALTS  267
OVOL1-Tetraodon    209  ERRNKLYVCEECGHTS-ASQDDLLVHLHSLHPNSHLLKGKTARRTG-GGGKAAMTS  262
OVOL1-Danio        185  ERRSKLYVCEECGHTA-STQDALLRHLHTEHPNSAFLRAKGARRHS-----PSLNG  234
OVOL2-Human        208  QRRDKLYVCEDCGYTG-PTQEDLYLHVNSAHPGSSFLK-KTSKKLA-----ALLQG  256
OVOL2-Mouse        207  QRRDKLYVCEDCGYTG-PTQEDLYLHVNSDHPGSTFLK-KTSKKLA-----ALMQN  255
OVOL2-Rat          207  QRRDKLYVCEDCGYTG-PTQEDLYLHVNSAHPGSTFLK-KTSKKLA-----ALMQN  255
OVOL2-Opossum      200  QRRDKLYVCEDCGYTG-PTQEDLYLHVNSAHPGSAFLK-KTSKKLA-----AILQT  248
OVOL2-Chicken      197  QRRDKLYVCEDCGYTG-PTQEDLYLHVSSVHPSSAFLK-KTSKKLA-----AVLQN  245
OVOL2-Zebrafinch   198  QRRDKLYVCEDCGYTG-PTQEDLYLHVSGIHPGSAFLK-KTSKKLA-----AVLQT  246
OVOL2-Anolelizard  208  QRRDKLYVCEDCGYTG-PTQEDLYMHISNIHPGSTLLK-KTSKKMT-----AVLQN  256
OVOL2-Frog         220  QRRDKLFVCEDCGYTG-PSQEDLYLHVYDSHPGSAFLK-KTTKKLA-----AILQT  268
OVOL3-Human        154  ERREKLHVCEDCGFTS-SRPDTYAQHRALHRAA-----------------------  185
OVOL3-Mouse        206  ERREKLHVCEDCGFT-----------------------------------------  220
OVOL3-Rat          191  ERREKLHVCEDCGFT-----------------------------------------  205
OVOL3b-Danio       188  QRRSKIFVCEDCGFTS-SRPDQYFLHVRQQHPGSPALR-RYYRKHM--LEGPGPHN  239
OVOL3b-Fugu         88  QRRSKIFVCEDCGYTS-SRPDEYFLHVRQCHPGSPALR-RYYRRQA----------  131
OVOL3b-Medaka      181  QRRSKIFVCEDCGYTS-SRPDEYFLHVRQRHPGSPALR-R----------------  218
OVOLBFL1            86  ERREKIYVCEECGHAT-MSPEEHLEHLKQVHPEN----------------------  118
OVOLBFL2            86  ERREKIYVCEECGHAT-MSPEEHLEHLKQVHPEN----------------------  118
OVOLNVE2            97  ERRDKMFVCEDCGITF-KDSPEFMKHVHELHPET----------------------  129
OVOLNVE1            90  ERRDKMFVCEDCGITFKENQAEYRQHVASHHP------------------------  121

                          β11
OVOL1-Human        256  TVTSLLQGSPHL----------  267
OVOL1-Mouse        256  TVTSLLQGSPHL----------  267
OVOL1-Rat          256  TVTSLLQGSPHL----------  267
OVOL1-Opossum      258  TVTALLQNNHHL----------  269
OVOL1-Stickleback  253  EGSSPGSPQGADSDDTTGSAGQ  274
OVOL1-Fugu         268  ALESPQSAESDISSGSTEQ---  286
OVOL1-Tetraodon    263  APESPQSAESDVSTGSADQ---  281
OVOL1-Danio        235  TKDDTSQPGSPLSLNSDDNAES  256
OVOL2-Human        257  KLTSAHQENTSLSEEEERK---  275
OVOL2-Mouse        256  KLTSPLQENSTLSEEEEKK---  274
OVOL2-Rat          256  KLTSPLQENSTLSEEEEKK---  274
OVOL2-Opossum      249  KLTSVLQRNAKLSEEDK-----  265
OVOL2-Chicken      246  KLSPVLQRNSKDDDKDE-----  262
OVOL2-Zebrafinch   247  KLSPVLQRNSKEDGKDE-----  263
OVOL2-Anolelizard  257  KLTS------------------  260
OVOL2-Frog         269  KITSVLQIKSTAQEEVEAE---  287
OVOL3-Human          -  ----------------------    -
OVOL3-Mouse          -  ----------------------    -
OVOL3-Rat            -  ----------------------    -
OVOL3b-Danio       240  ISPYMLYPSAGLYI--------  253
OVOL3b-Fugu          -  ----------------------    -
OVOL3b-Medaka        -  ----------------------    -
OVOLBFL1             -  ----------------------    -
OVOLBFL2             -  ----------------------    -
OVOLNVE2             -  ----------------------    -
OVOLNVE1             -  ----------------------    -
```
