# Supplementary material for: Molecular Phylogeny of OVOL Genes Illustrates a Conserved C2H2 Zinc Finger Domain Coupled by Hypervariable Unstructured Regions
Source: PLoS One. 2012 Jun 21;7(6):e39399. doi: 10.1371/journal.pone.0039399 (PMC3380836; doi:10.1371/journal.pone.0039399)
Supplement: Figure S2 — Similarities and differences among Drosophila OVOA-D and mouse OVOL1-OVOL3 using protein sequence alignment. Zinc finger motif is a highly conserved region (red shading). The presence of multiple stretches of the same amino acids are visible in this alignment in the N-terminal regions. (PDF) [file pone.0039399.s002.pdf]

Figure S2.

```
OVO-C-Dmelanogaster    1 --------------------------------------------------------------------------------
OVO-B-Dmelanogaster    1 MPKIFLIKNRLHQQQQRLLESQNLLQHKNQDDERLVPPLSPSGSGSGPSPTPTSQPPPEPQGQGQQVLGQVPDSDQQPLS
OVO-A-Dmelanogaster    1 --------------------------------------------------------------------------------
OVO-D-Dmelanogaster    1 --------------------------------------------------------------------------------
OVOL3-Mouse            1 --------------------------------------------------------------------------------
OVOL1-Mouse            1 --------------------------------------------------------------------------------
OVOL2-Mouse            1 --------------------------------------------------------------------------------

OVO-C-Dmelanogaster    1 -------------------------------------------------MNVNKNDLQNFAAELLQRLTPNTATTAQNNI
OVO-B-Dmelanogaster   81 LTRKRFHHRRHYFGQSRHSLDHLNQNQSPNPNANANPNQIQNPAELEVECATGQVQNENFAAELLQRLTPNTATTAQNNI
OVO-A-Dmelanogaster    1 --------------------------------------------------------------------------------
OVO-D-Dmelanogaster    1 --------------------------------------------------------------------------------
OVOL3-Mouse            1 --------------------------------------------------------------------------------
OVOL1-Mouse            1 --------------------------------------------------------------------------------
OVOL2-Mouse            1 --------------------------------------------------------------------------------

OVO-C-Dmelanogaster   32 VNNLVNNSRAATSVLATKDCTIENSPISIPKNQRAEDEEEQEDQEKEKPAEREREKSDERTEQVEKEERVEREEEEDDEV
OVO-B-Dmelanogaster  161 VNNLVNNSRAATSVLATKDCTIENSPISIPKNQRAEDEEEQEDQEKEKPAEREREKSDERTEQVEKEERVEREEEEDDEV
OVO-A-Dmelanogaster    1 --------------------------------------------------------------------------------
OVO-D-Dmelanogaster    1 --------------------------------------------------------------------------------
OVOL3-Mouse            1 --------------------------------------------------------------------------------
OVOL1-Mouse            1 --------------------------------------------------------------------------------
OVOL2-Mouse            1 --------------------------------------------------------------------------------

OVO-C-Dmelanogaster  112 DVGVEAPRPRFYNTGVVLTQAQRKEYPQEPKDLSLTIAKSSPASPHIHSDSESDSDSDGGCKLIVDEKPPLPVIKPLSLR
OVO-B-Dmelanogaster  241 DVGVEAPRPRFYNTGVVLTQAQRKEYPQEPKDLSLTIAKSSPASPHIHSDSESDSDSDGGCKLIVDEKPPLPVIKPLSLR
OVO-A-Dmelanogaster    1 --------------------------------------------------------------------------------
OVO-D-Dmelanogaster    1 --------------------------------------------------------------------------------
OVOL3-Mouse            1 --------------------------------------------------------------------------------
OVOL1-Mouse            1 --------------------------------------------------------------------------------
OVOL2-Mouse            1 --------------------------------------------------------------------------------

OVO-C-Dmelanogaster  192 LRSTPPPADQRPSPPPPRDPAPAVRCSVIQRAPQSQLPTSRAGFLLPPLDQLGPEQQEPIDYHVPKRRSPSYDSDEELNA
OVO-B-Dmelanogaster  321 LRSTPPPADQRPSPPPPRDPAPAVRCSVIQRAPQSQLPTSRAGFLLPPLDQLGPEQQEPIDYHVPKRRSPSYDSDEELNA
OVO-A-Dmelanogaster    1 --------------------------------------------------------------------------------
OVO-D-Dmelanogaster    1 --------------------------------------------------------------------------------
OVOL3-Mouse            1 --------------------------------------------------------------------------------
OVOL1-Mouse            1 --------------------------------------------------------------------------------
OVOL2-Mouse            1 --------------------------------------------------------------------------------

OVO-C-Dmelanogaster  272 RRLERARQVREARRRSTILAARVLLAQSQRLNPRLVRSLPGILAAAAGHGRNSSSSSGAAGQGFQSSGFGSQNSGSGSSS
OVO-B-Dmelanogaster  401 RRLERARQVREARRRSTILAARVLLAQSQRLNPRLVRSLPGILAAAAGHGRNSSSSSGAAGQGFQSSGFGSQNSGSGSSS
OVO-A-Dmelanogaster    1 --------------------------------------------------------------------------------
OVO-D-Dmelanogaster    1 --------------------------------------------------------------------------------
OVOL3-Mouse            1 --------------------------------------------------------------------------------
OVOL1-Mouse            1 --------------------------------------------------------------------------------
OVOL2-Mouse            1 --------------------------------------------------------------------------------

OVO-C-Dmelanogaster  352 GNQNAGSGAGSPGSGAGGGGGMGGGRDGRGNYGPNSPPTGALPPFYESLKSGQQSTASNNTGQSPGANHSHFNANPANFL
OVO-B-Dmelanogaster  481 GNQNAGSGAGSPGSGAGGGGGMGGGRDGRGNYGPNSPPTGALPPFYESLKSGQQSTASNNTGQSPGANHSHFNANPANFL
OVO-A-Dmelanogaster    1 ---------------------MGGGRDGRGNYGPNSPPTGALPPFYESLKSGQQSTASNNTGQSPGANHSHFNANPANFL
OVO-D-Dmelanogaster    1 ---------------------MGGGRDGRGNYGPNSPPTGALPPFYESLKSGQQSTASNNTGQSPGANHSHFNANPANFL
OVOL3-Mouse            1 -----------------------------------------MPRVFLVRS------------RRPQPP--NWSHLPDQL-
OVOL1-Mouse            1 -----------------------------------------MPRAFLVKK------------PCVSTCKRNWSELPDEE-
OVOL2-Mouse            1 -----------------------------------------MPKVFLVKR------------RSPGVSVRSWDELPDDK-

OVO-C-Dmelanogaster  432 QNAAAAAYIMSAGSGGGGCTGNGGGGASGPGGGPSANSGGGGGGGGGNGYINCGGVGGPNNSLDGNNLLNFASVSNYNES
OVO-B-Dmelanogaster  561 QNAAAAAYIMSAGSGGGGCTGNGGGGASGPGGGPSANSGGGGGGGGGNGYINCGGVGGPNNSLDGNNLLNFASVSNYNES
OVO-A-Dmelanogaster   60 QNAAAAAYIMSAGSGGGGCTGNGGGGASGPGGGPSANSGGGGGGGGGNGYINCGGVGGPNNSLD----------------
OVO-D-Dmelanogaster   60 QNAAAAAYIMSAGSGGGGCTGNGGGGASGPGGGPSANSGGGGGGGGGNGYINCGGVGGPNNSLDGNNLLNFASVSNYNES
OVOL3-Mouse           25 ---------------------------------------------RGDAYV-----------------------------
OVOL1-Mouse           27 ---------------------------------------------RGEIYV-----------------------------
OVOL2-Mouse           27 ---------------------------------------------RADTYI-----------------------------

OVO-C-Dmelanogaster  512 NSKFHNHHHHHQHNNNNNNNGGQTSMMGHPFYGGNPSAYGIILKDEPDIEYDEAKIDIGTFAQNIIQATMGSSGQFNASA
OVO-B-Dmelanogaster  641 NSKFHNHHHHHQHNNNNNNNGGQTSMMGHPFYGGNPSAYGIILKDEPDIEYDEAKIDIGTFAQNIIQATMGSSGQFNASA
OVO-A-Dmelanogaster  124 -------------------------------------AYGIILKDEPDIEYDEAKIDIGTFAQNIIQATMGSSGQFNASA
OVO-D-Dmelanogaster  140 NSKFHNHHHHHQHNNNNNNNGGQTSMMGHPFYGGNPSAYGIILKDEPDIEYDEAKIDIGTFAQNIIQATMGSSGQFNASA
OVOL3-Mouse           31 -----------------------------------PGSWLPLLHPGP---------------------------------
OVOL1-Mouse           33 -----------------------------------PVSLGFCPPQPY---------------------------------
OVOL2-Mouse           33 -----------------------------------PVSLGCLLRDPP---------------------------------
```

```
OVO-C-Dmelanogaster  592 YEDAIMSDLASSGQCPNGAVDPLQFTATLMLSSQTDHLLEQLSDAVDLSSFLQRSCVDDEESTSPRQDFELVSTPSLTPD
OVO-B-Dmelanogaster  721 YEDAIMSDLASSGQCPNGAVDPLQFTATLMLSSQTDHLLEQLSDAVDLSSFLQRSCVDDEESTSPRQDFELVSTPSLTPD
OVO-A-Dmelanogaster  167 YEDAIMSDLASSGQCPNGAVDPLQFTATLMLSSQTDHLLEQLSDAVDLSSFLQRSCVDDEESTSPRQDFELVSTPSLTPD
OVO-D-Dmelanogaster  220 YEDAIMSDLASSGQCPNGAVDPLQFTATLMLSSQTDHLLEQLSDAVDLSSFLQRSCVDDEESTSPRQDFELVSTPSLTPD
OVOL3-Mouse           43 ------SSTQSQGSVFSGC---------------------------------------EEDSKKG---------------
OVOL1-Mouse           45 ------REPEASVAEPPSC--PLALDMSLR---------------------------DSSYSVAP---------------
OVOL2-Mouse           45 ------EDCRSDGGSSSGC---------------------------------------SSSAGEP---------------

OVO-C-Dmelanogaster  672 SVTPVEQHNTNTTQLDVLHENLLTQLTHNIVRGGSNQQQQHHQQHGVQQQQQQQHSVQQQQQHNVQQQHGVQQQHVQQQP
OVO-B-Dmelanogaster  801 SVTPVEQHNTNTTQLDVLHENLLTQLTHNIVRGGSNQQQQHHQQHGVQQQQQQQHSVQQQQQHNVQQQHGVQQQHVQQQP
OVO-A-Dmelanogaster  247 SVTPVEQHNTNTTQLDVLHENLLTQLTHNIVRGGSNQQQQHHQQHGVQQQQQQQHSVQQQQQHNVQQQHGVQQQHVQQQP
OVO-D-Dmelanogaster  300 SVTPVEQHNTNTTQLDVLHENLLTQLTHNIVRGGSNQQQQHHQQHGVQQQQQQQHSVQQQQQHNVQQQHGVQQQHVQQQP
OVOL3-Mouse           63 ---------------------------------------------------------------------GGSVSLTSQDC
OVOL1-Mouse           75 ---------------------------------------------------------------------G---PCVVAQL
OVOL2-Mouse           65 ---------------------------------------------------------------------GGAESSSSPRA

OVO-C-Dmelanogaster  752 PPSYQHATRGLMMQQQPQHGGYQQQAAIMSQQQQQLLSQQQQQSHHQQQQQQQHAAAYQQHNIYAQQQQQQQQQHHQQQQ
OVO-B-Dmelanogaster  881 PPSYQHATRGLMMQQQPQHGGYQQQAAIMSQQQQQLLSQQQQQSHHQQQQQQQHAAAYQQHNIYAQQQQQQQQQHHQQQQ
OVO-A-Dmelanogaster  327 PPSYQHATRGLMMQQQPQHGGYQQQAAIMSQQQQQLLSQQQQQSHHQQQQQQQHAAAYQQHNIYAQQQQQQQQQHHQQQQ
OVO-D-Dmelanogaster  380 PPSYQHATRGLMMQQQPQHGGYQQQAAIMSQQQQQLLSQQQQQSHHQQQQQQQHAAAYQQHNIYAQQQQQQQQQHHQQQQ
OVOL3-Mouse           74 PSLWAHNYRVAKTEQDPA--------------------------------------------------------------
OVOL1-Mouse           83 P-------------------------------------------------------------------------------
OVOL2-Mouse           76 P-------------------------------------------------------------------------------

OVO-C-Dmelanogaster  832 QQQHHHFHHQQQQQPQPQSHHSHHHGHGHDNSNMSLPSPTAAAAAAAAAAAAAAAAAAAAHLQRPMSSSSSSGGTNSSNSSG
OVO-B-Dmelanogaster  961 QQQHHHFHHQQQQQPQPQSHHSHHHGHGHDNSNMSLPSPTAAAAAAAAAAAAAAAAAAAAHLQRPMSSSSSSGGTNSSNSSG
OVO-A-Dmelanogaster  407 QQQHHHFHHQQQQQPQPQSHHSHHHGHGHDNSNMSLPSPTAAAAAAAAAAAAAAAAAAAAHLQRPMSSSSSSGGTNSSNSSG
OVO-D-Dmelanogaster  460 QQQHHHFHHQQQQQPQPQSHHSHHHGHGHDNSNMSLPSPTAAAAAAAAAAAAAAAAAAAAHLQRPMSSSSSSGGTNSSNSSG
OVOL3-Mouse           92 ---------------------------GHTN-------------------------------PYTLWQSSQGTLTS----
OVOL1-Mouse           84 -----------------------------------------------------SEDVSHLTDPQSRDQGFLRTKMKVTLG
OVOL2-Mouse           77 -------------EPETPELHDAQGTDGH---------------------------LAAMQRPVARS-------------

OVO-C-Dmelanogaster  912 GSSNSPLLDANAAAAAAAALLDTKPLIQS---------------------------------------------------
OVO-B-Dmelanogaster 1041 GSSNSPLLDANAAAAAAAALLDTKPLIQS---------------------------------------------------
OVO-A-Dmelanogaster  487 GSSNSPLLDANAAAAAAAALLDTKPLIQSVSNPIGQPLNTQSQQQKQGQQITLMKTTRYTEFVEMVSMDVTVKPELFSEL
OVO-D-Dmelanogaster  540 GSSNSPLLDANAAAAAAAALLDTKPLIQSVSNPIGQPLNTQSQQQKQGQQITLMKTTRYTEFVEMVSMDVTVKPELFSEL
OVOL3-Mouse          110 --------------------------------------------------------------------------------
OVOL1-Mouse          111 DSPNGDL-------------------------------------------------------------------------
OVOL2-Mouse          104 --------------------------------------------------------------------------------

OVO-C-Dmelanogaster  941 --------------------------------------------------------------------------------
OVO-B-Dmelanogaster 1070 --------------------------------------------------------------------------------
OVO-A-Dmelanogaster  567 KPEMTEITAEELTLEAETTAAAAAAAAAAAAAATTTSATEGTQVLAAAPAPLSSGRKLRGRAKAVAYGSTMITLISTLKSS
OVO-D-Dmelanogaster  620 KPEMTEITAEELTLEAETTAAAAAAAAAAAAAATTTSATEGTQVLAAAPAPLSSGRKLRGRAKAVAYGSTMITLISTLKSS
OVOL3-Mouse          110 --------------------------------------------------------------------------------
OVOL1-Mouse          118 --------------------------------------------------------------------------------
OVOL2-Mouse          104 --------------------------------------------------------------------------------

OVO-C-Dmelanogaster  941 -----------------------------------------------LGLPPDLQLEFVNGGHGIKNPLAVENAHGGHHR
OVO-B-Dmelanogaster 1070 -----------------------------------------------LGLPPDLQLEFVNGGHGIKNPLAVENAHGGHHR
OVO-A-Dmelanogaster  647 PEVPATKTVHRTTLRSLATAAAATAAGLLAPSPTVSVLNESKVLQRRLGLPPDLQLEFVNGGHGIKNPLAVENAHGGHHR
OVO-D-Dmelanogaster  700 PEVPATKTVHRTTLRSLATAAAATAAGLLAPSPTVSVLNESKVLQRRLGLPPDLQLEFVNGGHGIKNPLAVENAHGGHHR
OVOL3-Mouse          110 --------------------------------------------------------------------------------
OVOL1-Mouse          118 --------------------------------------------------------------------------------
OVOL2-Mouse          104 ------------------------------------------------------KIKETTG-------------------

OVO-C-Dmelanogaster  974 IRNIDCIDDLSKHGHHSQHQQQQGSPQQQNMQQSVQQQSVQQQQSLQQQQQQQHHQHHSNSSASSNASSHGSAEALCMGS
OVO-B-Dmelanogaster 1103 IRNIDCIDDLSKHGHHSQHQQQQGSPQQQNMQQSVQQQSVQQQQSLQQQQQQQHHQHHSNSSASSNASSHGSAEALCMGS
OVO-A-Dmelanogaster  727 IRNIDCIDDLSKHGHHSQHQQQQGSPQQQNMQQSVQQQSVQQQQSLQQQQQQQHHQHHSNSSASSNASSHGSAEALCMGS
OVO-D-Dmelanogaster  780 IRNIDCIDDLSKHGHHSQHQQQQGSPQQQNMQQSVQQQSVQQQQSLQQQQQQQHHQHHSNSSASSNASSHGSAEALCMGS
OVOL3-Mouse          110 --------------------------------------------------------------------------------
OVOL1-Mouse          118 --------------------------------------------------------------------------------
OVOL2-Mouse          111 --------------------------------------------------------------------------------

OVO-C-Dmelanogaster 1054 SGGANEDSSSGNNKFVCRVCMKTFSLQRLLNRHMKCHSDIKRYLCTFCGKGFNDTFDLKRHTRTHTGVRPYKCNLCEKSF
OVO-B-Dmelanogaster 1183 SGGANEDSSSGNNKFVCRVCMKTFSLQRLLNRHMKCHSDIKRYLCTFCGKGFNDTFDLKRHTRTHTGVRPYKCNLCEKSF
OVO-A-Dmelanogaster  807 SGGANEDSSSGNNKFVCRVCMKTFSLQRLLNRHMKCHSDIKRYLCTFCGKGFNDTFDLKRHTRTHTGVRPYKCNLCEKSF
OVO-D-Dmelanogaster  860 SGGANEDSSSGNNKFVCRVCMKTFSLQRLLNRHMKCHSDIKRYLCTFCGKGFNDTFDLKRHTRTHTGVRPYKCNLCEKSF
OVOL3-Mouse          110 -------APKGPGTLGCPLCPKAFPLQRMLTRHLKCHSPARRHVCHYCGKGFHDAFDLKRHMRTHTGIRPFRCGACGKAF
OVOL1-Mouse          118 --------------FTCHICQKSFTHQRMLNRHMKCHNDVKRHLCTYCGKGFNDTFDLKRHVRTHTGVRPYKCSLCDKAF
OVOL2-Mouse          111 -------TCDNSVIHNCDLCGKSFRLQRMLNRHLKCHNQVKRHLCTFCGKGFNDTFDLKRHVRTHTGIRPYKCEVCNKAF
```

```
OVO-C-Dmelanogaster 1134 TQRCSLESHCQKVHSVQHQYAYKERRAKMYVCEECGHTTCEPEVHYLHLKNNHPFSPALLKFYDKRHFKFTNSQFANNLL
OVO-B-Dmelanogaster 1263 TQRCSLESHCQKVHSVQHQYAYKERRAKMYVCEECGHTTCEPEVHYLHLKNNHPFSPALLKFYDKRHFKFTNSQFANNLL
OVO-A-Dmelanogaster  887 TQRCSLESHCQKVHSVQHQYAYKERRAKMYVCEECGHTTCEPEVHYLHLKNNHPFSPALLKFYDKRHFKFTNSQFANNLL
OVO-D-Dmelanogaster  940 TQRCSLESHCQKVHSVQHQYAYKERRAKMYVCEECGHTTCEPEVHYLHLKNNHPFSPALLKFYDKRHFKFTNSQFANNLL
OVOL3-Mouse          183 TQRCSLEAHLAKVHGQPASYAYRERREKLHVCEDCGFT------------------------------------------
OVOL1-Mouse          184 TQRCSLESHLKKIHGVQQKYAYKERRAKLYVCEECGCTSESQEGHVLHLKERHPDSP-LLRKTSKKVAVALQNTVTSLLQ
OVOL2-Mouse          184 TQRCSLESHLKKIHGVQQQYAYKQRRDKLYVCEDCGYTGPTQEDLYLHVNSDHPGST-FLKKTSKKLAALMQNKLTSPLQ

OVO-C-Dmelanogaster 1214 GQLPMPVHN---
OVO-B-Dmelanogaster 1343 GQLPMPVHN---
OVO-A-Dmelanogaster  967 GQLPMPVHN---
OVO-D-Dmelanogaster 1020 GQLPMPVHN---
OVOL3-Mouse              ------------
OVOL1-Mouse          263 GSPHL-------
OVOL2-Mouse          263 ENSTLSEEEEKK
```
