# Supplementary figures and images for: Molecular Phylogeny of OVOL Genes Illustrates a Conserved C2H2 Zinc Finger Domain Coupled by Hypervariable Unstructured Regions
Source: PLoS One. 2012 Jun 21;7(6):e39399. doi: 10.1371/journal.pone.0039399 (PMC3380836; doi:10.1371/journal.pone.0039399)

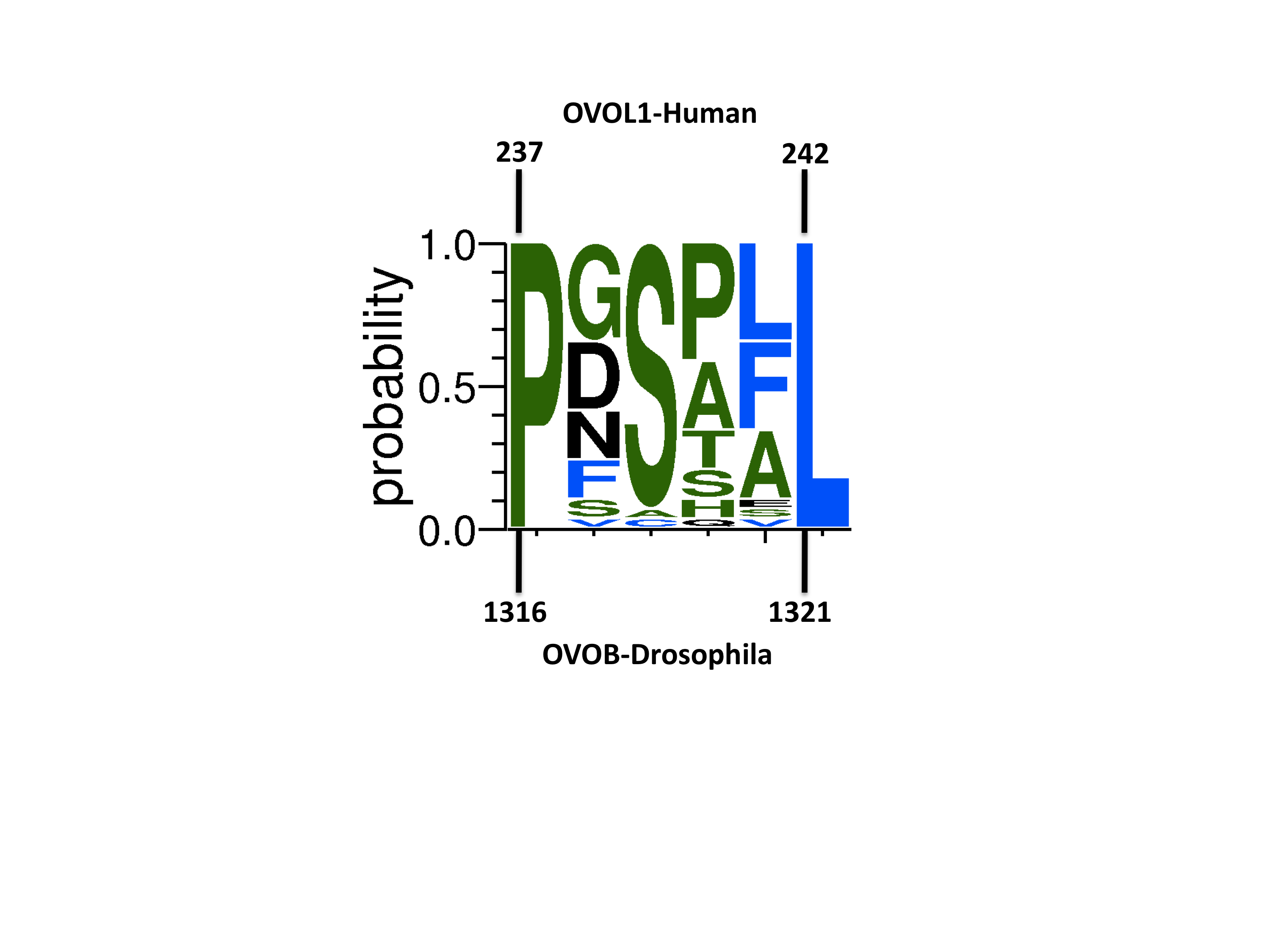

Supplement: Figure S3 — A highly conserved motif is found immediately after the fourth C2H2 motif present in the majority of OVOL proteins from metazoan origin. We generated this sequence logo using WebLogo 3.0 [81]. (PNG) [file pone.0039399.s003.png]
